# Characteristics associated with SF-36 in alpha-1 antitrypsin deficiency-associated COPD: a cross-sectional analysis

**DOI:** 10.1186/s12890-024-02953-7

**Published:** 2024-03-18

**Authors:** Radmila Choate, Kristen E. Holm, Robert A. Sandhaus, David M. Mannino, Charlie Strange

**Affiliations:** 1 University of Kentucky College of Public Health, Lexington, Kentucky United States; 2 Department of Medicine, National Jewish Health, Denver, Colorado United States; 3 AlphaNet, Inc, Coral Gables, Florida, United States; 4 University of Kentucky College of Medicine, Lexington, Kentucky United States; 5 Division of Pulmonary, Critical Care, Allergy and Sleep Medicine, Medical University of South Carolina, Charleston, South Carolina United States

**Keywords:** COPD, Alpha-1 antitrypsin deficiency, Quality of life

## Abstract

**Background:**

Generic measures of health-related quality of life (HRQoL), such as the 36-Item Short Form Survey (SF-36), are widely used in assessing chronic conditions. These tools have an advantage over disease-specific instruments, as they allow comparisons across different health conditions and with the general population. In alpha-1 antitrypsin deficiency (AATD)-associated chronic obstructive pulmonary disease (COPD), HRQoL research remains scarce. This cross-sectional study evaluates the factors associated with HRQoL in a cohort of patients with AATD-associated COPD.

**Methods:**

Our study included participants of AlphaNet (2008-2019), a health management organization for people with AATD in the US who are prescribed augmentation therapy. Norm-based SF-36 scores for the mental and physical component summary scores (MCS and PCS, mean of 50 ± 10 in the general US population) and 8 individual scales were evaluated. Individuals with lung disease and data available on ≥1 measurement on any SF-36 scale and clinically relevant characteristics such as modified Medical Research Council (mMRC) scale, exacerbation frequency, productive cough, and use of oxygen were included in these analyses. Generalized linear regression models were fit to examine the association of baseline characteristics with MCS and PCS scores. Age, sex, regular use of oxygen, exacerbation frequency, mMRC, and productive cough were included in these models.

**Results:**

Participants (*n*=4398, mean age 57.6 [SD=10.6] years, 45.4% female) had a mean MCS score of 51.2 ± 10.8 and PCS of 36.3 ± 9.8. The average mMRC score was 2.4 ± 1.3, and 56.4% had 2 or more exacerbations per year. Overall, the physical component of SF-36 was more severely impacted compared to the mental component. In multivariable regression analyses, PCS scores were significantly associated with exacerbation frequency, mMRC, regular use of oxygen, and productive cough; MCS was associated with age, sex, exacerbation frequency, mMRC, and productive cough.

**Conclusions:**

These findings demonstrate that patient-perceived physical health is significantly impaired in this cohort of people with AATD-associated COPD compared to mental health. Longitudinal studies are needed to evaluate the change in physical and mental health status over time in this population.

**Supplementary Information:**

The online version contains supplementary material available at 10.1186/s12890-024-02953-7.

## Introduction

Individuals with alpha-1 antitrypsin deficiency (AATD) are genetically predisposed to premature loss of lung function and development of emphysema, particularly if they smoke tobacco or are exposed to environmental toxins [1, 2]. Those with severely deficient AATD genotypes, including ZZ, ZNull, NullNull, and SZ, often carry a higher risk of lung disease progression [3]. Unfortunately, AATD-associated lung disease is often underdiagnosed [2, 4], impacting day-to-day activities as symptoms of chronic obstructive pulmonary disease (COPD), such as shortness of breath and cough develop, leading to worsened quality of life [5].

Health-related quality of life (HRQoL) is a broad measure of self-perceived disease burden in people with lung disease. Research indicates that those with AATD-associated COPD experience increased dyspnea at a younger age compared to non-AATD COPD [6], resulting in earlier and more significant impairment in health status. AATD-associated COPD is treated with symptom management and weekly intravenous augmentation therapy of purified plasma-derived alpha-1 antitrypsin; however, these therapies do not cure the disease. Thus, improving HRQoL in this population is highly important. Various instruments, both disease-specific (e.g., the Saint George’s Respiratory Questionnaire [SGRQ]) and generic (e.g., the 36-item Short-Form Health Survey [SF-36]), have been developed and validated to assess the quality of life impairment in people with lung disease [7, 8]. Research indicates that the COPD-specific SGRQ is highly correlated with clinical measures and pulmonary symptoms distinguishing between levels of COPD severity [8]. On the other hand, generic HRQoL instruments, like SF-36, offer a more comprehensive assessment of health, evaluating the general impact of the disease on various aspects of well-being, including mental health and physical functioning [9]. Moreover, generic HRQoL assessment tools allow comparisons across different health conditions and with the general population [10]. Studies show that SF-36 is a valid instrument for measuring HRQoL in people with COPD [11]. Although HRQoL is not commonly used as a primary outcome measure in COPD clinical trials, many therapeutic interventions in COPD are designed to improve individuals’ quality of life [12]. Research on HRQoL in populations with AATD-associated COPD remains limited, with findings often variable and inconsistent regarding the burden of disease [13]. Assessing factors influencing the deterioration of HRQoL in people living with AATD will improve our understanding of the natural history and progression of the disease and aid in the development of interventions to stabilize and improve HRQoL in this population. Therefore, the present study aims to evaluate the association between HRQoL and patient- and disease-specific characteristics within a large cohort of individuals with AATD-associated lung disease.

## Methods

### Participants and procedures

Participants in AlphaNet, a not-for-profit health management organization for individuals with alpha-1 antitrypsin deficiency (AATD) in the United States who are prescribed augmentation therapy, were included in this study [14]. Individuals were diagnosed with AATD and prescribed augmentation therapy by their own physicians and subsequently referred to AlphaNet. The present cross-sectional analyses included data collected by AlphaNet Coordinators via structured telephone interviews between 2008 and 2019.

**Fig. 1:**
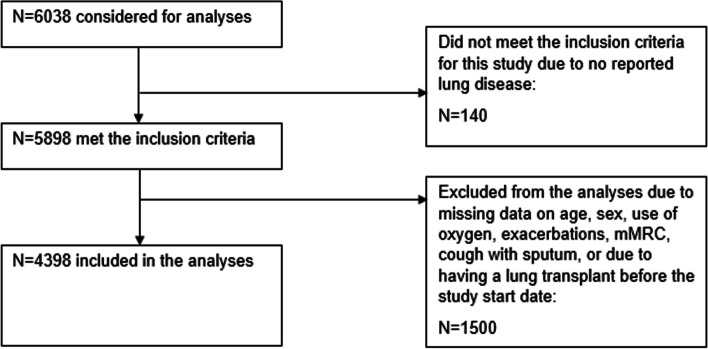
Study flow diagram

*Inclusion and exclusion criteria.* AlphaNet participants with baseline data on age, sex, clinically relevant variables such as dyspnea, frequency of exacerbations, regular use of oxygen, and productive cough in the past year, as well as data on one or more components of SF-36 were included in this study. Participants with unavailable data on key baseline variables of interest, not reporting having lung disease, and those who had a lung transplant before the study start date were excluded from the analyses (Fig. 1).

### Measures

*HRQoL* was measured using SF-36 version 2 administered during a separate telephone interview scheduled after the baseline assessment had been completed. SF-36 is a generic patient-reported outcome measure designed to quantify health status in clinical practice and research [15]. SF-36 is one of the most widely used generic HRQoL instruments in COPD research. This measure consists of 8 domains describing limitations in health status: (1) physical functioning, (2) role limitations due to physical problems, (3) bodily pain, (4) general health perceptions, (5) vitality, (6) social functioning, (7) role limitations due to emotional problems, and (8) general mental health. Each subscale score ranges from 0 to 100, with higher scores reflecting better HRQoL. The subscales are summarized into 2 summary components: the physical component summary (PCS) and the mental component summary (MCS). PCS and MCS are norm-based scores with a mean of 50 (SD=10) describing the average US general population health status in 1998.

*Dyspnea* was assessed using a modified Medical Research Council (mMRC) dyspnea scale [16]. The mMRC scale ranges from grade 0 to 4 and measures the degree of disability that dyspnea poses on individuals’ daily activities. Data on the frequency of exacerbations, productive cough, and use of oxygen were collected using standardized questionnaires.

These analyses were conducted under the approval of the University of Kentucky Institutional Review Board (#43435).

### Statistical analysis

Data were summarized using frequencies and proportions for categorical variables and means, standard deviations (SD), medians, and interquartile ranges (IQR) for continuous variables. We used analysis of variance (ANOVA) to compare means of SF-36 subscales and summary scores across the categories of baseline variables of interest: age, sex, mMRC, frequency of exacerbations in the prior year, oxygen use, and productive cough in the two years before enrollment. These variables were selected *a priori* based on clinical relevance in existing COPD literature. We fit unadjusted and adjusted generalized linear regression models to examine the association between SF-36 summary scores and baseline variables of interest. Univariate models assessed the association between each variable and PCS and MCS scores. Multivariable analyses included as predictors only the variables that had a statistically significant association in univariate models. The goodness of fit of the final models was assessed using adjusted R^2^. Sensitivity analyses were conducted to compare individuals who provided SF-36 measurements within the first year after the baseline assessment to those evaluated after 1 year. The significance level for all analyses was set at 0.05. We used SAS software, Version 9.4 to perform the analyses.

## Results

Overall, 4398 AlphaNet participants were included in this study (Fig. 1). The average age of the participants was 57.6 (SD=10.6) years; 45.4% were women, and 96.1% were non-Hispanic White. Severe AATD genotypes (ZZ, ZNull, or NullNull) were prevalent in this cohort (Table 1). Most participants (56.4%) experienced two or more exacerbations in the past year, and 44.0% had daily productive cough for at least 3 months each year over the past 2 years (Table 1). The mean mMRC score was 2.4 (SD=1.3), and 49.6% of all participants had mMRC scores of 3 or 4.

**Table 1:** Demographic and clinical characteristics of the study population and SF-36 summary scores, *n*=4398

	n (%)	Mental Component Summary (MCS) score^a^	Physical Component Summary (PCS) score^a^
		mean (SD)	mean (SD)
Age, years			
<50	1010 (22.96)	48.15 (11.57)	35.65 (10.05)
50-65	2355 (53.55)	51.27 (10.64)	36.30 (9.83)
>65	1033 (23.49)	53.97 (9.37)	37.10 (9.29)
Sex			
Female	1996 (45.38)	51.58 (10.52)	36.38 (9.91)
Male	2402 (54.62)	50.87 (10.95)	36.31 (9.64)
mMRC^b^			
0	404 (9.19)	54.25 (8.36)	45.48 (9.27)
1	986 (22.42)	53.63 (8.96)	41.87 (8.75)
2	826 (18.78)	51.92 (10.42)	36.78 (8.29)
3	950 (21.60)	50.94 (10.89)	33.64 (8.06)
4	1232 (28.01)	47.90 (11.99)	30.63 (8.05)
Frequency of exacerbations in the past year			
0	1001 (22.76)	53.99 (9.07)	40.32 (9.56)
1	915 (20.80)	52.89 (9.89)	38.39 (9.48)
2 or more	2482 (56.43)	49.43 (11.35)	33.97 (9.25)
Regular use of oxygen in the past year			
No	2193 (49.86)	51.67 (10.46)	39.33 (9.77)
Yes	2205 (50.14)	50.71 (11.04)	33.36 (8.80)
Daily productive cough for at least 3 months each year over the past 2 years			
No	2462 (55.98)	52.35 (10.20)	37.96 (9.76)
Yes	1936 (44.02)	49.72 (11.27)	34.27 (9.38)
Alpha1 variant			
ZZ	2305 (62.16)	51.89 (10.29)	37.23 (9.96)
ZNull	31 (0.84)	52.11 (11.31)	36.91 (9.64)
NullNull	12 (0.32)	53.17 (10.10)	34.54 (9.99)
FF & FZ	31 (0.84)	50.80 (9.59)	35.38 (9.88)
SZ	382 (10.30)	51.24 (10.53)	36.50 (9.73)
MZ	733 (19.77)	50.43 (11.44)	35.05 (9.42)
Other^c^	214 (5.77)	50.19 (11.26)	34.62 (9.80)
Data available *n*=3708			

^a^MCS and PCS have a mean of 50 (±10) in the general US population. Higher scores indicate better HRQoL

^b^modified Medical Research Council scale: 0--I only get breathless with strenuous exercise; 1 -I get short of breath when hurrying on level ground or walking up a slight hill; 2 -On level ground, I walk slower than people of the same age because of breathlessness or have to stop for breath when walking; 3 -I stop for breath after walking about 100 yards or after a few minutes on level ground; 4 -I am too breathless to leave the house, or I am breathless when dressing

^c^includes FM, FS, IM, IS, IZ, MS, and other

The majority of participants (*n*=3365, 77%) completed SF-36 within one year of baseline, with an average of 130.7 (SD=108.1) days between baseline assessment and SF-36. In the 1033 individuals who completed SF-36 more than one year after baseline (23.5% of the sample), the average time between the baseline and SF-36 was 829.1 (SD=471.2) days. Participants in the two groups did not differ by age, mMRC, or presence of productive cough and had similar PCS scores. The group with more than a year between the two assessment dates had a greater proportion of males, lower use of oxygen, higher number of exacerbations, and better MCS scores. (Supplemental Table 1).

**Fig. 2:**
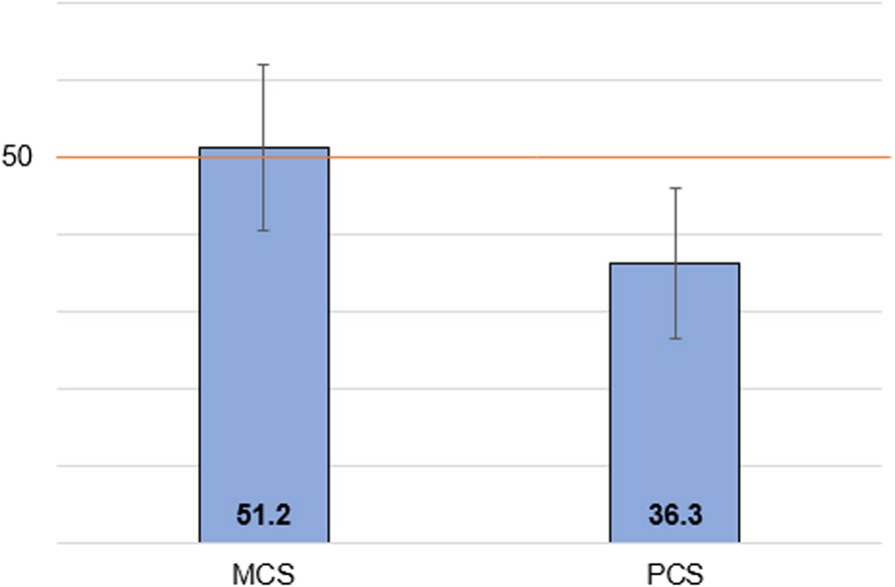
Baseline SF-36 summary component score means in the overall cohort, *n*=4398. Note: SF-36 summary scores are norm-based, with an average of 50 (SD=10) for the general US population. Error bars reflect standard deviations

**Fig. 3:**
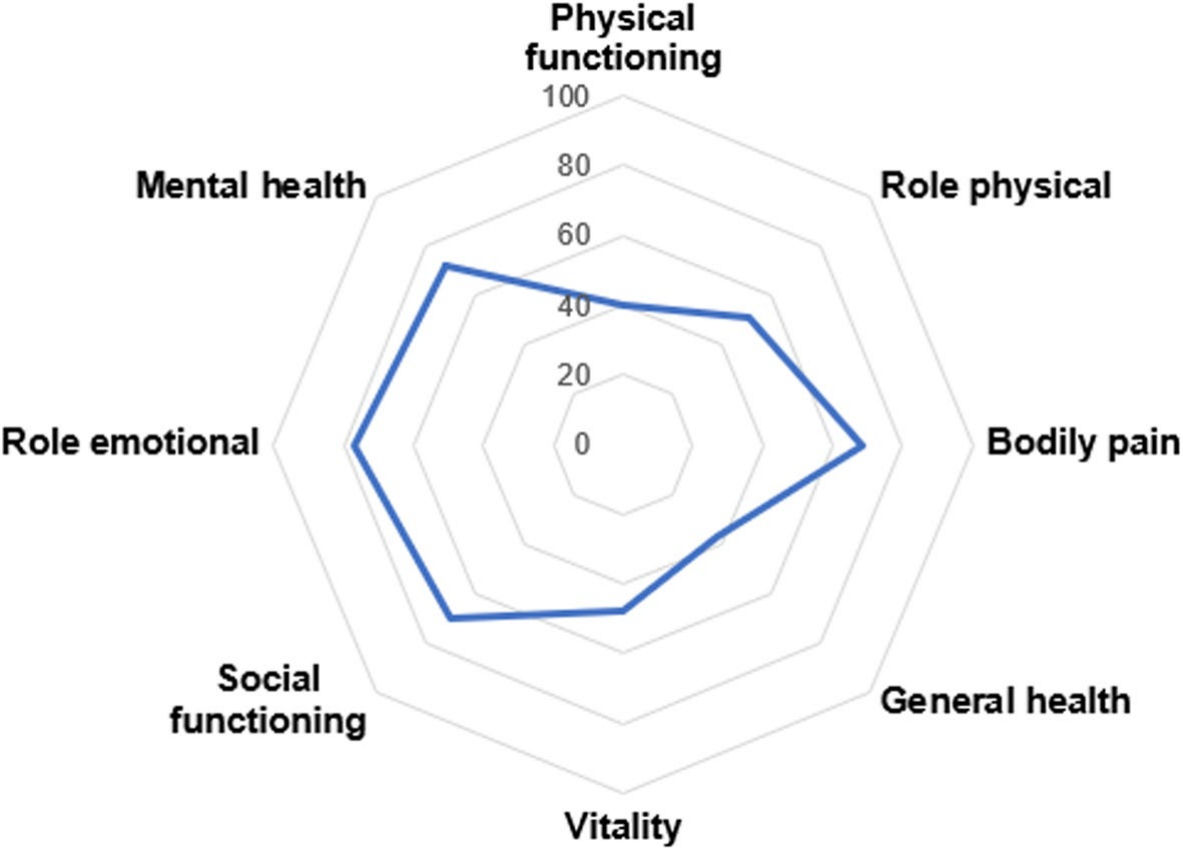
SF-36 subscale scores in the overall cohort, *n*=4398. Note: SF-36 subscale scores range from 0-100, with higher scores indicating better health

As demonstrated in Fig. 2, physical health in this cohort was markedly lower than in the general US population, with the mean PCS score of 36.3 (SD=9.8). In contrast, mental health was comparable with the general US population, with the mean MCS score of 51.2 (SD= 10.8). SF-36 subscale scores for physical functioning, role physical, and general health and vitality were the most impacted domains, with mean scores in the lower half of the scale range (Fig. 3).

**Fig. 4:**
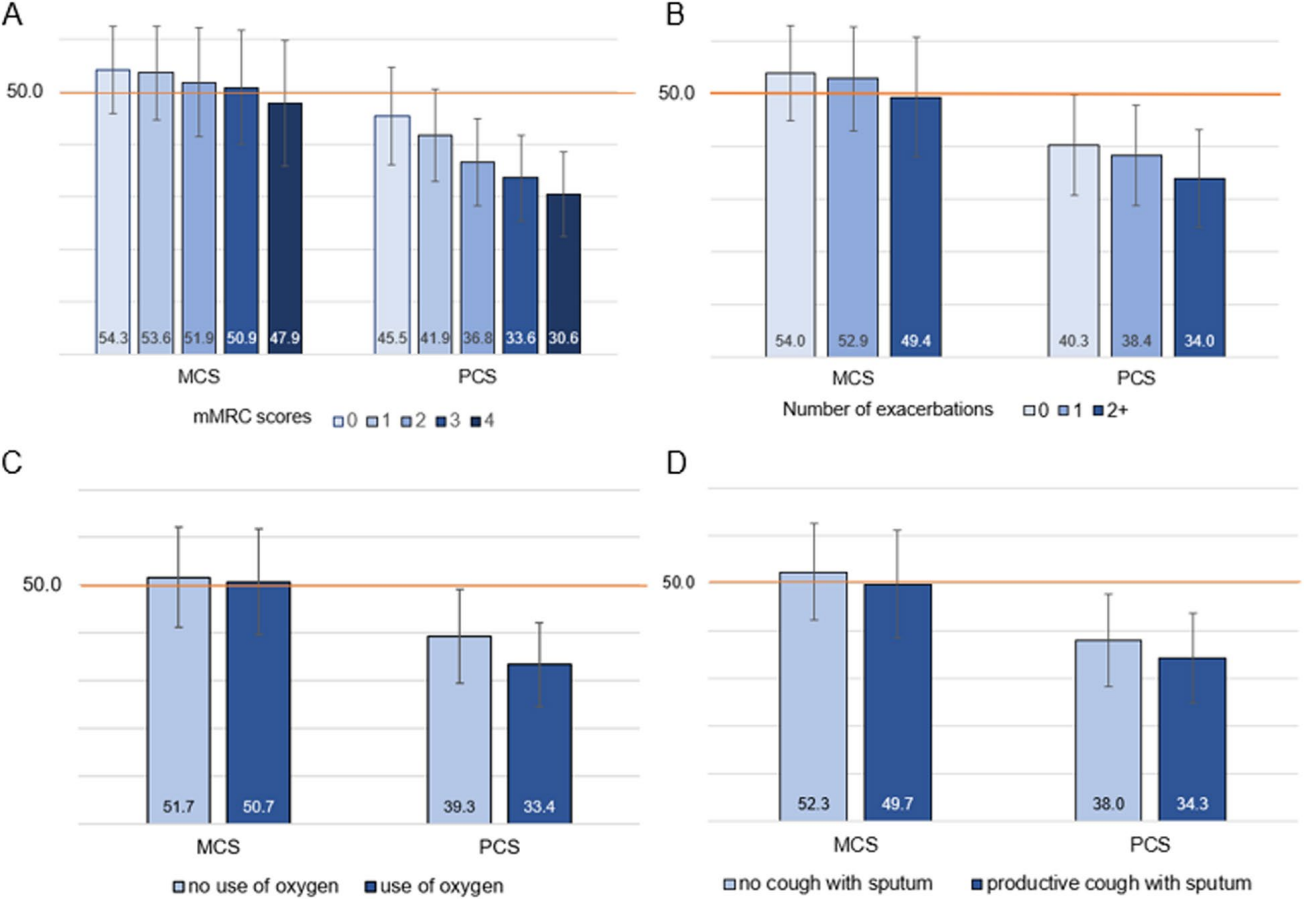
SF-36 summary scores by clinical characteristics- **A**) mMRC score, **B**) number of exacerbations at baseline, **C**) use of oxygen and **D**) having productive cough with sputum. Note: MCS, mental summary score, and PCS, physical summary score: mean 50 (SD=10) in the general US population. *P*<0.0001 for comparison of mean MCS and PCS scores by mMRC, frequency of exacerbations, and productive cough. *P*=0.0034 for comparison of mean MCS and <.0001 for mean PCS scores by use of oxygen

Both summary component scores, MCS and PCS, showed a significant negative dose-response relationship with mMRC scores, reflecting worse health status with greater dyspnea (*p*<.0001 for both component summary scores) (Fig. 4A). Across all dyspnea grades, physical health had greater impairment than mental health. Similarly, both MCS and PCS mean scores demonstrated a dose-response association with the frequency of exacerbations at baseline (Supplemental Figure 1). Higher number of exacerbations reported at baseline was significantly associated with worse MCS and PCS scores (*p*<.0001 for both comparisons) (Fig. 4B). Subscales reflecting general health, vitality, and physical functioning were the most impacted domains by exacerbations (Supplemental Figure 1). We observed significantly better physical health (*p*<.0001) and better mental health (*p*=0.0034) in people who do not report using oxygen regularly, compared to those who do (Fig. 4C). Those on regular oxygen had markedly lower physical functioning and role limitations due to physical problems compared to those not on oxygen (Supplemental Figure 2). Similarly, individuals reporting daily productive cough for at least 3 months each year over the past 2 years had significantly lower physical and mental health status compared to those with no cough (*p*<.0001 for both scores comparison) (Fig. 4D). General health and physical functioning were the most impaired domains in those with productive cough (Supplemental Figure 2).

**Fig. 5:**
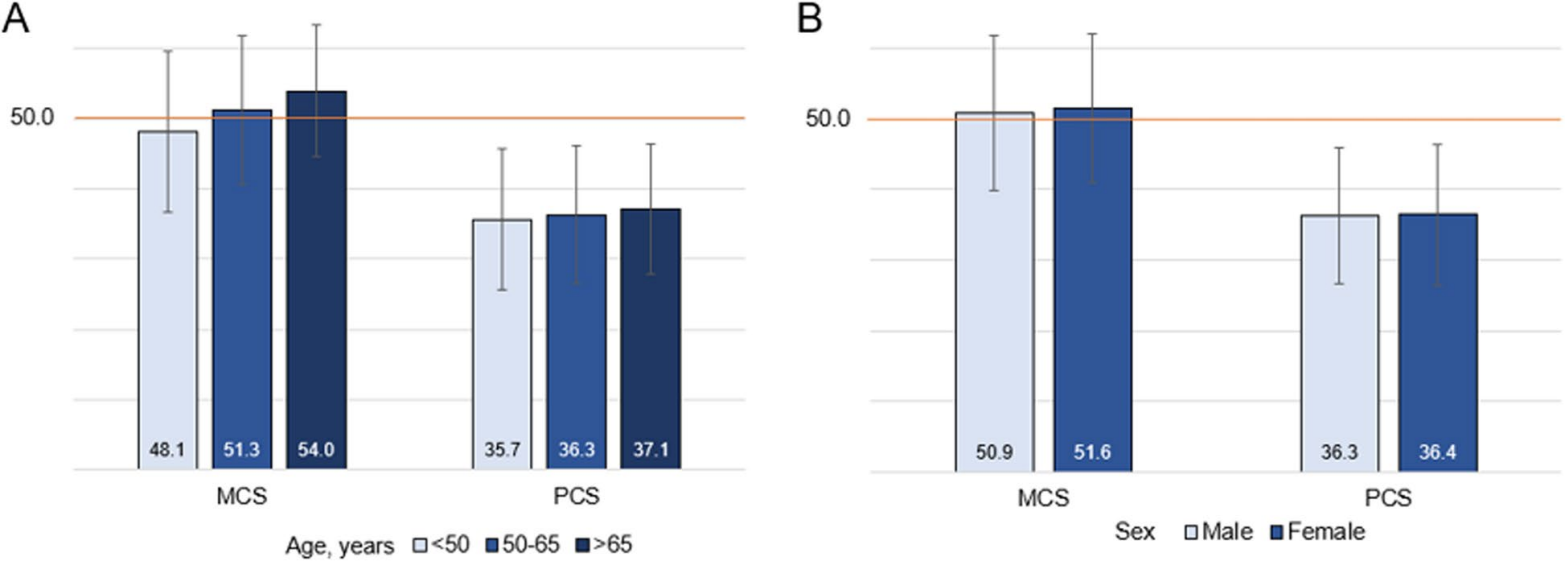
SF-36 summary scores by demographic characteristics – **A**) age and **B**) sex. Note: MCS, mental summary score, and PCS, physical summary score: mean 50 (SD=10) in the general US population. P<0.0001 for comparison of mean MCS scores and *P*=0.0040 for comparison of mean PCS scores across age groups. No significant difference in MCS and PCS scores by sex

As shown in Fig. 5, age had a positive dose-response relationship with MCS and PCS; older age was associated with better quality of life (*p*<.0001 for MCS and *p*=0.0040 for PCS). There was no difference in physical health status by sex and slightly better mental health status in women (*p*=0.0318) (Fig. 5). Older participants had better SF-36 scores in all subscales except for physical functioning, which was not different across the age groups. There was no difference in mental or physical subscale scores between males and females. (Supplemental Figure 3). In addition, we observed comparable MCS and PCS scores across Alpha1 variants with no dose-response relationship by genotype severity (Table 1).

In univariate analyses, all *a priori* selected variables were significantly associated with MCS and PCS scores, except for sex, which was not a significant determinant of PCS score (Table 2). Multivariable regression models for MCS and PCS included predictors significantly associated with each score in univariate analyses (Table 3). In the adjusted model, all variables except for regular use of oxygen were significantly associated with MCS score. PCS score was associated with mMRC score, frequency of exacerbations, regular use of oxygen, and productive cough. Age was not significantly associated with PCS score adjusted for other covariates in the model. Overall, based on the adjusted regression coefficients, the effects of covariates were generally attenuated compared to the univariate models (Tables 2 and 3).

**Table 2:** Univariate associations between demographic and clinical characteristics and MCS and PCS of SF-36

	MCS^a^			PCS^a^		
	Unadjusted coefficient	95% CI	*p*-value	Unadjusted coefficient	95% CI	*p*-value
Age, years						
<50	REF			REF		
50-65	3.12	(2.33; 3.91)	<0.0001	0.65	(-0.08; 1.38)	0.0797
>65	5.82	(4.89; 6.75)	<0.0001	1.45	(0.59; 2.31)	0.0009
Sex						
Male	REF			REF		
Female	0.71	(0.06; 1.36)	0.0318	0.07	(-0.51; 0.66)	0.8043
mMRC^b^						
0	REF			REF		
1	-0.62	(-1.86; 0.61)	0.3203	-3.61	(-4.59; -2.63)	<0.0001
2	-2.34	(-1.86; -1.07)	0.0003	-8.70	(-9.70; -7.69)	<0.0001
3	-3.32	(-4.55; -2.08)	<0.0001	-11.85	(-12.83; -10.86)	<0.0001
4	-6.36	(-7.56; -5.16)	<0.0001	-14.85	(-15.81; -13.90)	<0.0001
Frequency of exacerbations in the past year						
0	REF			REF		
1	-1.10	(-2.06; -0.14)	0.0243	-1.92	(-2.78; -1.08)	<0.0001
2 or more	-4.56	(-5.35; -3.78)	<0.0001	-6.34	(-7.03; -5.64)	<0.0001
Regular use of oxygen in the past year						
No	REF			REF		
Yes	-0.96	(-1.61; -0.32)	0.0034	-5.97	(-6.53; -5.42)	<0.0001
Daily productive cough for at least 3 months each year over the past 2 years						
No	REF			REF		
Yes	-2.63	(-3.27; -1.99)	<0.0001	-3.69	(-4.27; -3.11)	<0.0001

^a^MCS (Mental Component Summary score) and PCS (Physical Component Summary score) have a mean of 50 (±10) in the general US population. Higher scores indicate better HRQoL

^b^modified Medical Research Council scale: 0 -I only get breathless with strenuous exercise; 1 -I get short of breath when hurrying on level ground or walking up a slight hill; 2 -On level ground, I walk slower than people of the same age because of breathlessness or have to stop for breath when walking; 3 -I stop for breath after walking about 100 yards or after a few minutes on level ground; 4 -I am too breathless to leave the house, or I am breathless when dressing

**Table 3:** Multivariable associations between demographic and clinical characteristics and MCS and PCS of SF-36

	MCS^a^			PCS^a^		
	Adjusted coefficient	95% CI	*p*-value	Adjusted coefficient	95% CI	*p*-value
Age, years						
<50	REF			REF		
50-65	2.59	(1.82; 3.39)	<.0001	0.20	(-0.41; 0.81)	0.5228
>65	4.74	(3.82; 5.67)	<.0001	0.43	(-0.31; 1.16)	0.2526
Sex						
Male	REF			--		
Female	0.79	(0.18; 1.41)	0.0117	--		
mMRC^b^						
0	REF			REF		
1	-0.45	(-1.65; 0.76)	0.4662	-3.04	(-4.0; -2.09)	<.0001
2	-1.75	(-3.00; -0.50)	0.063	-7.25	(-8.25; -6.26)	<.0001
3	-2.40	(-3.66; -1.15)	0.0002	-9.73	(-10.72; -8.74)	<.0001
4	-5.07	(-6.32; 3.82)	<.0001	-12.03	(-13.02; -11.04)	<.0001
Frequency of exacerbations in the past year						
0	REF			REF		
1	-0.38	(-1.32; 0.56)	0.4265	-0.46	(-1.20; 0.28)	0.2233
2 or more	-2.59	(-3.40; -1.78)	<.0001	-3.03	(-3.67; -2.38)	<.0001
Regular use of oxygen in the past year						
No	REF			REF		
Yes	0.30	(-0.38; 0.97)	0.3871	-2.62	(-3.15; -2.09)	<.0001
Daily productive cough for at least 3 months each year over the past 2 years						
No	REF			REF		
Yes	-1.21	(-1.85; -0.56)	0.0003	-1.37	(-1.88; -0.86)	<.0001

^a^MCS (Mental Component Summary score) and PCS (Physical Component Summary score) have a mean of 50 (±10) in the general US population. Higher scores indicate better HRQoL

^b^modified Medical Research Council scale: 0--I only get breathless with strenuous exercise; 1 -I get short of breath when hurrying on level ground or walking up a slight hill; 2 -On level ground, I walk slower than people of the same age because of breathlessness or have to stop for breath when walking; 3 -I stop for breath after walking about 100 yards or after a few minutes on level ground; 4 -I am too breathless to leave the house, or I am breathless when dressing. Adjusted R^2^ for the MCS model is 0.09. Adjusted R^2^ for the PCS model is 0.30. Sex was not included in the multivariable model for PCS as not significantly associated in univariate analysis

## Discussion

Our study of HRQoL in a cohort of people living with AATD-associated COPD demonstrated a substantial disease impact on the physical aspect of their lives, but no demonstrable impact on mental well-being when compared to population norms. We identified several disease aspects of COPD as determinants of lower self-perceived quality of life, including dyspnea and frequent exacerbations, as well as markers of disease severity, including regular use of oxygen and chronic productive cough.

Living with COPD impacts an individual’s physical and social daily activities and well-being. With disease progression, dyspnea is often the primary symptom leading to the reduction in regular physical activities and social interactions and hence impacts the quality of one’s life. Literature suggests that dyspnea scores tend to be closely correlated with general health status in COPD [17, 18]. Dyspnea is one of the main foci of the disease-specific HRQoL instruments, such as SGRQ, and has been shown to correlate with worse health status in people with COPD [19, 20]. In our study, we used a disease-agnostic, generic instrument and demonstrated similar associations of worse HRQoL in those with greater levels of dyspnea. Our findings are consistent with a study that found a strong association between dyspnea and the physical health component of SF-36 and a moderate association with its mental health component [21].

Research shows that frequent acute exacerbations are associated with long-term impact on HRQoL in patients with COPD and AATD-associated lung disease [22, 23]. Consistent with other studies, our analyses showed consistently worse mental and physical health in people experiencing exacerbations, predominantly impacting physical health domains. Moreover, the strongest association with low HRQoL was observed in frequent exacerbators reporting 2 or more exacerbations per year.

Cough with sputum production is often the primary reason for seeking medical care in people with COPD [24]. In our study, individuals reporting having daily productive cough for at least 3 months each year over the past 2 years had significantly worse physical and mental HRQoL compared to those not experiencing this symptom. SF-36 subscale scores were consistently lower in those with productive cough across all domains. Several studies support our findings of association between productive cough and HRQoL [25–27]. Regular use of oxygen in our study was considered a proxy measure of disease severity and was prevalent in over a half of the participants. Our findings demonstrated significant impairment in physical functioning and general health in those on regular oxygen. Previous research showing worse physical functioning-related HRQoL in people with COPD on long-term oxygen treatment support our results [28].

In this study, younger participants reported less favorable HRQoL, particularly limitations due to physical problems, general health, and vitality. This paradoxical finding may be associated with higher life expectations in younger adults and the impact of AATD-associated COPD on these expectations of health and vitality [29]. Other studies support our findings and report an inverse association between age and HRQoL in people with COPD [30] and AATD-associated lung disease [31]. We found similar associations in our earlier work examining HRQoL in people with AATD using a disease-specific instrument, SGRQ [29]. Our analyses demonstrated that in this cohort of people with AATD-associated lung disease, males and females had comparable experiences with their physical quality of life as well as specific domains of mental health. The overall summary component of mental health showed a moderately better perception in women. These findings are supported by previous research showing no difference in HRQoL between sexes [32] or greater decline in HRQoL in men with COPD [33]. In contrast, other studies report greater impairment of HRQoL in women using SGRQ [34] and SF-36 instruments [25].

Our results indicate that the multivariable model fit for PCS that included age, mMRC, frequency of exacerbation, use of oxygen, and productive cough explained a moderate 30% of the variability in the score mean. However, the multivariable model fit for MCS that included the above-mentioned variables as well as sex was able to explain only 9% of the MCS score variability. The remaining variance may be associated with other predictors of mental health not included in the present analyses. The results from an earlier study show that in people with COPD, MCS score was associated with dyspnea, depression, use of antidepressants, education, and daytime sleepiness [35]. These variables explained 35% of the MCS variability in that study. Depression and anxiety were identified as important predictors of low self-perceived quality of life in several other COPD studies [25]. Interestingly, research indicates that people with AATD-associated COPD do not report higher anxiety and depression symptoms compared to those with non-AATD COPD [6]. Other authors indicate that anxiety and depression are common in the AATD population and may contribute to worsened health status [36]. Since our study findings demonstrate that only a very small part of mental health is determined by lung disease-specific factors, there is an unmet need for psychological, behavioral, and cognitive assessment in this population of people with AATD-associated lung disease.

### Strength and limitations

Some of the strengths of this study include the large sample size in these analyses that allowed for robust evaluation of HRQoL and included many COPD disease-specific factors. Data collected from AlphaNet participants, individuals with AATD-associated lung disease who participate in a disease management program, allowed us to utilize real-world evidence in characterizing people living with AATD and their perceived physical and mental health.

The findings of this study should be evaluated in light of several limitations. First, data on potential determinants of HRQoL, such as pulmonary function, depression and anxiety evaluations were not available for analyses and may have limited the interpretation of the results. The data used in this study is self-reported by participants and may be subject to recall and reporting bias. The findings of this study may not be generalizable to all individuals with AATD-associated lung disease as the participants of this study receive tailored disease-specific education and resources from AlphaNet. Finally, this is a cross-sectional study; hence no causal inferences can be made.

## Conclusions

Patient-perceived physical health is significantly impaired in this cohort with AATD-associated lung disease, whereas mental health status is comparable to the general US population. Several disease-specific factors are associated with worse HRQoL and need to be taken into consideration to optimize the quality of life of people living with AATD. Further longitudinal studies are needed to examine the changes in SF-36 physical and mental health scores over time and the factors associated with these changes.

### Supplementary Information

**Supplementary Material 1.** 

## Data Availability

The datasets generated and analysed during the current study are not publically available but may be available from the corresponding author upon reasonable request.

## Abbreviations

AATD: Alpha-1 antitrypsin deficiency
COPD: Chronic obstructive pulmonary disease
HRQoL: Health-related quality of life
SGRQ: Saint George’s Respiratory Questionnaire
SF-36: 36-item Short-Form Health Survey
PCS: Physical component summary
MCS: Mental component summary
mMRC: Modified Medical Research Council
SD: Standard deviation
IQR: Interquartile range
ANOVA: Analysis of variance
