# Supplementary material for: Characteristics associated with SF-36 in alpha-1 antitrypsin deficiency-associated COPD: a cross-sectional analysis
Source: BMC Pulm Med. 2024 Mar 18;24:138. doi: 10.1186/s12890-024-02953-7 (PMC10949668; doi:10.1186/s12890-024-02953-7)
Supplement: Supplementary file 1 — Supplementary Material 1. [file 12890_2024_2953_MOESM1_ESM.docx]

SUPPLEMENTAL MATERIALS

**Supplemental Table 1**. Baseline characteristics of the cohort stratified by <1 year and ≥1 year between baseline assessment and SF-36 measurement, n=4398

|  | <1 year  N=3365 (76.5%) | ≥1year  N=1033  (23.5%) | p-value |
| --- | --- | --- | --- |
| Age, yrs, mean (SD)  Median (IQR) | 57.5 (10.5)  58 (50-65) | 57.8 (10.8)  59 (51-66) | 0.3448 |
| Female | 1561 (46.4) | 435 (42.1) | 0.0157 |
| Regular use of oxygen in the past year | 1755 (52.2) | 450 (43.6) | <0.0001 |
| Frequency of exacerbations in the past year |  |  | 0.0088 |
| 2 or more times  Once  Never | 1858 (55.2)  728 (21.6)  779 (23.2) | 354 (60.4)  187 (18.1)  222 (21.5) |  |
| mMRC^1^, mean (SD)  Median (IQR) | 2.4 (1.3)  3 (1-4) | 2.3 (1.3)  2 (1-4) | 0.1266 |
| Cough with sputum | 1755 (43.9) | 450 (43.6) | 0.7055 |
| SF-36 MCS, mean (SD)  Median (IQR) | 50.9 (10.9)  54 (44-59) | 52.3 (10.4)  55 (46-60) | 0.0003 |
| SF-36 PCS, mean (SD)  Median (IQR) | 36.4 (9.7)  36 (29-43) | 36.0 (9.9)  36 (28-44) | 0.2741 |

Data presented in frequencies and proportions unless otherwise noted.

^1^ modified Medical Research Council scale: 0 -I only get breathless with strenuous exercise; 1 -I get short of breath when hurrying on level ground or walking up a slight hill; 2 -On level ground, I walk slower than people of the same age because of breathlessness or have to stop for breath when walking; 3 -I stop for breath after walking about 100 yards or after a few minutes on level ground; 4 -I am too breathless to leave the house, or I am breathless when dressing.

**Supplemental Figure 1**. SF-36 subscale mean scores by A) mMRC and B) exacerbation frequency at baseline


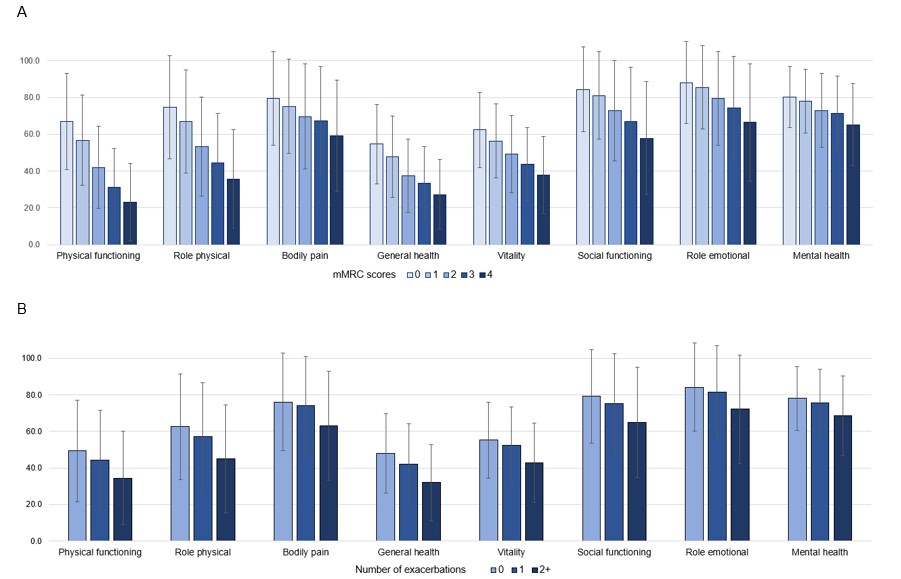


Note: Higher scores indicate better HRQoL

**Supplemental Figure** **2**. SF-36 subscale mean scores by A) use of oxygen and B) having productive cough at baseline


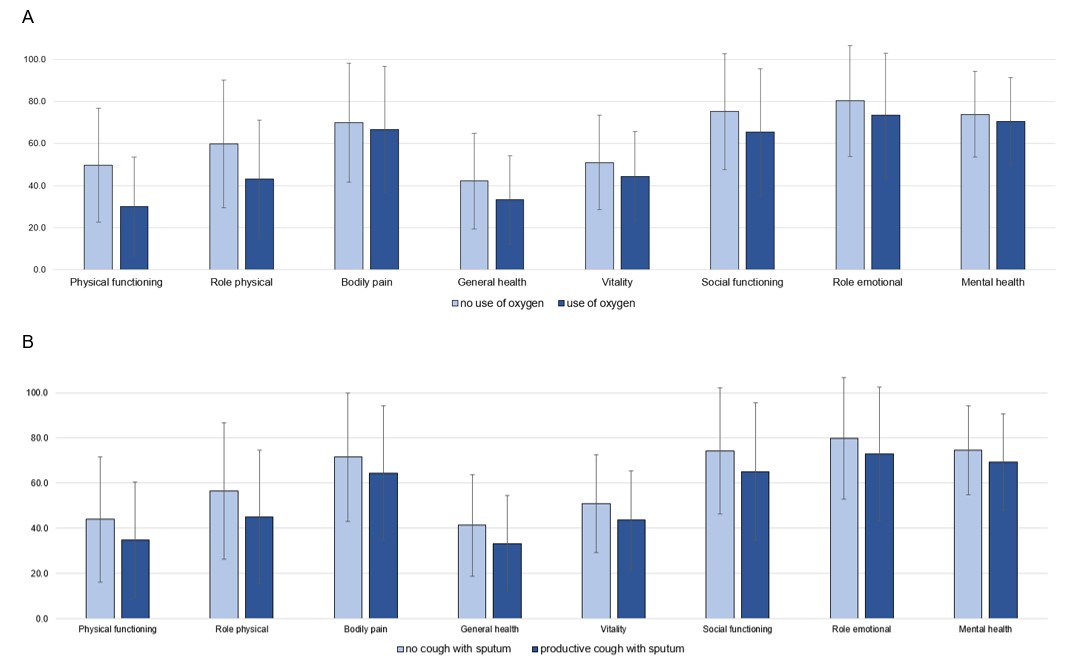


Note: Higher scores indicate better HRQoL

**Supplemental Figure 3**. SF-36 subscale mean scores by A) sex and B) age


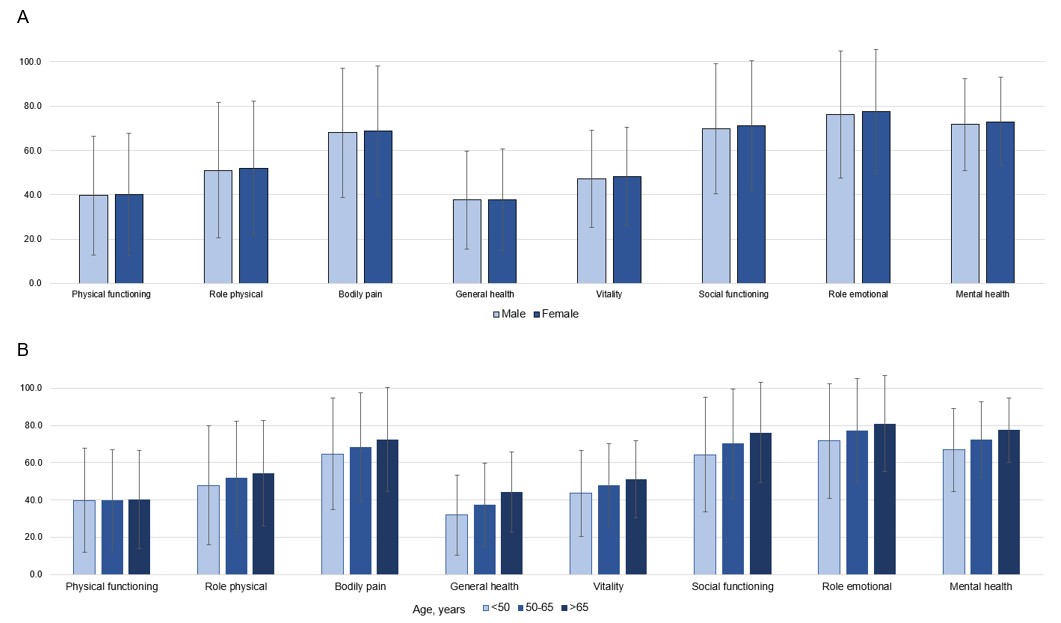


Note: Higher scores indicate better HRQoL
